# Satellite cell activity is differentially affected by contraction mode in human muscle following a work-matched bout of exercise

**DOI:** 10.3389/fphys.2014.00485

**Published:** 2014-12-11

**Authors:** Robert D. Hyldahl, Ty Olson, Tyson Welling, Logan Groscost, Allen C. Parcell

**Affiliations:** Department of Exercise Sciences, Brigham Young UniversityProvo, UT, USA

**Keywords:** eccentric, concentric, resistance exercise, stem cell, MyoD, Pax7

## Abstract

Optimal repair and adaptation of skeletal muscle is facilitated by resident stem cells (satellite cells). To understand how different exercise modes influence satellite cell dynamics, we measured satellite cell activity in conjunction with markers of muscle damage and inflammation in human skeletal muscle following a single work- and intensity-matched bout of eccentric (ECC) or concentric contractions (CON). Participants completed a single bout of ECC (*n* = 7) or CON (*n* = 7) of the knee extensors. A muscle biopsy was obtained before and 24 h after exercise. Functional measures and immunohistochemical analyses were used to determine the extent of muscle damage and indices of satellite cell activity. Cytokine concentrations were measured using a multiplexed magnetic bead assay. Isokinetic peak torque decreased following ECC (*p* < 0.05) but not CON. Greater histological staining of the damage marker Xin was observed in muscle samples of ECC vs. CON. Tenasin C immunoreactivity increased 15 fold (*p* < 0.01) following ECC and was unchanged following CON. The inflammatory cytokines interferon gamma-induced protein 10 (IP-10) and monocyte chemotactic protein 1 (MCP-1) increased pre- to post-ECC (4.26 ± 1.4 vs. 10.49 ± 5.8 pg/ml, and 3.06 ± 0.7 vs. 6.25 ± 4.6 pg/ml, respectively; *p* < 0.05). There was no change in any cytokine post-CON. Satellite cell content increased 27% pre- to post-ECC (0.10 ± 0.031 vs. 0.127 ± 0.041, respectively; *p* < 0.05). There was no change in satellite cell number in CON (0.099 ± 0.027 vs. 0.102 ± 0.029, respectively). There was no fiber type-specific satellite cell response following either exercise mode. ECC but not CON resulted in an increase in MyoD positive nuclei per myofiber pre- to post-exercise (*p* < 0.05), but there was no change in MyoD DNA binding activity in either condition. In conclusion, ECC but not CON results in functional and histological evidence of muscle damage that is accompanied by increased satellite cell activity 24 h post-exercise.

## Introduction

The unique regenerative and adaptive properties of skeletal muscle are largely under-lied by the action of muscle stem cells called satellite cells. Satellite cells are normally quiescent cells, identified initially by there distinct sublaminar location in the extracellular matrix (ECM) of skeletal muscle fibers (Mauro, 1961). In response to a sufficient stimulus (i.e., muscle injury or exercise), satellite cells exit their quiescent state (activation), proliferate, migrate to areas of damage, and fuse to the surrounding post-mitotic skeletal muscle (Yin et al., 2013). The activation and subsequent differentiation of satellite cells is facilitated by the expression of key transcription factors known as the myogenic regulatory factors (MRF; i.e, MyoD, Myf5, and myogenin). Studies suggest that optimal repair of injured skeletal muscle is dependent on a healthy and active pool of satellite cells (McCarthy et al., 2011; Murphy et al., 2011). Furthermore, growth of mechanically overloaded muscle is limited in satellite cell depleted animals (Fry et al., 2014), and it appears that in humans, individuals with a large satellite cell pool have a greater propensity for hypertrophic adaptations following exercise (Petrella et al., 2008). Therefore, investigations of the mechanisms that underlie satellite cell responsiveness to exercise are important.

The primary role of satellite cells is the repair of damaged muscle. Indeed, regeneration of muscle damage in satellite cell depleted animals is severely compromised (Murphy et al., 2011). It is also thought that satellite cells play a role in the growth and adaptation of muscle to normal physiological stress (i.e., exercise). However, their contribution in this regard is much more poorly understood. Previous studies have shown that the performance of resistance exercise and damage-inducing eccentric exercise results in activation of satellite cells and augmentation of the satellite cell pool in both younger and older men and women (Dreyer et al., 2006; Cermak et al., 2013; Leenders et al., 2013; Verdijk et al., 2014). In some cases, resistance training is also accompanied by increased myonuclear content, suggesting satellite cell fusion to myofibers (Petrella et al., 2008). Studies have likewise shown that aerobic exercise training promotes expansion of the satellite cell pool in the loaded muscles of animals and humans (Joanisse et al., 2013; Shefer et al., 2013). Thus, it is generally accepted that mechanical load induces satellite cell activity. Nevertheless, questions related to the intensity, frequency and mode of muscle action necessary to induce significant activity and expansion of the satellite cell pool have not been addressed. Given the importance of satellite cell activity to skeletal muscle health through the lifespan, an important, yet unanswered question relates to the influence of contraction mode on satellite cell activity. Eccentric (ECC) and concentric (CON) modes of exercise often result in a divergent physiological response. For example, it has been recognized for more than half a century that force production per metabolic cost is significantly higher in ECC vs. CON (Abbott et al., 1952), which contributes to differences in mitochondrial and metabolic outcomes with ECC and CON training (Isner-Horobeti et al., 2014). Furthermore, unaccustomed ECC has been widely reported to result in acute muscle damage, whereas CON, in most cases, does not (Faulkner et al., 1993). More recently, differences in intracellular signaling responses between the two contraction modes have been described (Vissing et al., 2013). Thus, it is plausible to speculate that their respective influence on satellite cell proliferation is correspondingly affected differentially.

To provide insight into this question, we devised an experimental paradigm whereby human subjects performed a single bout of either eccentric or concentric muscle contractions that were matched for total work volume and intensity (i.e., maximal effort). The primary objectives of the study were to assess (1) functional and histological markers of muscle damage, and (2) satellite cell content and activation status before and 24 h following a single bout of either eccentric or concentric contractions. Because eccentric but not concentric contractions are associated with a transient damage response, the repair of which appears to be the primary function of satellite cells, we hypothesized that eccentric contractions would promote a greater satellite cell response than concentric contractions.

## Materials and methods

### Subjects

Fourteen untrained young men were recruited for this study and randomized to perform a bout of concentric (CON) or eccentric (ECC) contractions (*n* = 7 per group). Based on an assessment of previously published literature, a sample size of 14 subjects (7 individuals per group) was determined to be large enough to detect differences in satellite cell proliferation 24 h post-eccentric exercise (Dreyer et al., 2006; O'Reilly et al., 2008). Rather than using opposite limbs to perform each contraction mode in a cross-over design, we used a between subject analysis and group design to avoid the potential confounding variable of a contralateral repeated bout effect (Xin et al., 2014). Since the number of subjects was relatively small, a homogenous subject population was chosen, restricting parameters that were likely to influence the muscle's response to exercise such as age, muscle training and sex. All subjects underwent a routine health questionnaire and screening. Subjects were informed of all procedures and potential risks and signed a written informed consent document approved by the Brigham Young University Institutional Review Board. Subjects had no prior history of musculoskeletal injury of the lower extremity and refrained from participating in new physical activities or taking oral or topical analgesics for the duration of the study.

### Study design

The study consisted of six laboratory visits. Subjects reported to the laboratory on their first day and began by receiving a familiarization session on the Biodex System 4 dynamometer (Biodex Medical Systems, Shirley, NY). Also on the first visit, baseline muscle strength and soreness were assessed and a biopsy sample was taken from the *m. vastus lateralis* that was randomized to be the non-exercised leg. The biopsy served as the pre-exercise control. Subjects reported back to the lab 24 h later to perform the respective concentric or eccentric exercise protocol. Twenty-four hours after the completion of the exercise protocol, subjects returned to the laboratory and underwent a muscle biopsy of the *m. vastus lateralis* on the exercised leg. Muscle strength and soreness were also assessed at this visit. Subjects returned to the laboratory every 24 h for the next 3 days to have their muscle strength and soreness assessed. Studies in rodent, and to more limited extent, humans suggest that MRF expression increases 24 h following exercise (McKay et al., 2012; Macaluso et al., 2013; Yin et al., 2013). Likewise, although measurable increases in satellite cell content most likely peak at later time points (i.e., 48–72 h post-exercise), increases in satellite cell content in human muscle has been widely reported 24 h following eccentric exercise (Dreyer et al., 2006; O'Reilly et al., 2008; Cermak et al., 2013). Thus, we chose to take the muscle biopsy 24 h post-exercise to facilitate the assessment of changes in both satellite cell content and MRF activity.

### Exercise protocol

Subjects performed a single unilateral bout of either maximal concentric or eccentric contractions of the knee extensors with a randomized leg on a Biodex System 4 dynamometer (Biodex Medical Systems, Shirley, NY). Subjects in both groups performed multiple sets of concentric or eccentric contractions until approximately 40 kJ of work was achieved. Sets consisted of approximately 2 kJ of work with a 1 min rest between sets. During each repetition, subjects to maximally resist (eccentric execisers) or maximally “kick” (concentric exercisers) the lever arm. Consistent verbal encouragement was provided throughout each repetition. Due to the higher force production of eccentric contractions, subjects in the ECC group did significantly fewer total muscle contractions through the bout of exercise (Table 1). However, in order to minimize the discrepancy in total number of contractions needed to reach 40 kJ for the two contraction modes, we used a slower speed for the lower force producing concentric contractions. Concentric contractions were performed at a rate of 60°·sec^−1^ and eccentric contractions were performed at 120°·sec^−1^. For eccentric contractions, subjects resisted as the lever pulled their partially extended leg from 40° of knee flexion (where 0° is full extension) to 115° of knee flexion, a total range of motion (ROM) of 75°. A similar range has been used in several previous studies to induce muscle damage and is based on the work of Beaton et al. (2002; Cermak et al., 2013). For the concentric contractions, limits to the dynamometer were designated at the ends of each subject's ROM. Subjects were told to perform the exercise at a self-selected ROM. Subjects exercised between a ROM of approximately 90° of knee flexion to 20° of knee flexion, a total ROM similar to the total ECC ROM, but without the longer muscle lengths. In both cases, subjects were verbally encouraged to provide a maximal effort.

**Table 1:** **Anthropometric and exercise performance data**.

**Contraction mode**	**Age (years)**	**Height (cm)**	**Weight (kg)**	**Work performed (kJ)**	**# Repetitions**
ECC (*n* = 7)	22.6 ± 2.1	180.4 ± 4.1	73.3 ± 16.4	40.8 ± 0.8	196.1 ± 37.2^*^
CON (*n* = 7)	23.5 ± 1.1	181.7 ± 6.9	82.8 ± 16.9	39.2 ± 2.3	350.9 ± 222.0

* *Indicates significant difference between ECC and CON (p < 0.05)*.

### Muscle biopsies

One percutaneous needle biopsy was taken from the *m. vastus lateralis* of each leg. The first biopsy was taken from the non-exercised leg prior to the exercise intervention. This biopsy sample served as the pre-exercise control. A second biopsy was taken from the exercised leg 24 h following the intervention, as this time point represented an optimal time to measure changes in both satellite cell proliferation and activity of the early MRF MyoD. Under local anesthesia (Lidocaine), a small incision was made into the skin and fascia, and the biopsy needle was inserted into the muscle. A small core of tissue (~100 mg) was withdrawn. Following withdrawal of the tissue sample it was separated from any fatty tissue. The lean tissue sample was then divided into multiple 25–50 mg portions. Portions that were to be homogenized and used for protein isolation were immediately frozen in liquid nitrogen. Portions of tissue designated for sectioning and microscopic analysis were mounted on a cork with tragacanth gum and frozen in isopentane cooled in liquid nitrogen to avoid freeze fracture.

### Maximal force producing capacity

Maximal isokinetic strength of the knee extensor muscles was assessed on the Biodex dynamometer (Biodex Medical Systems, Shirley, NY). Subjects completed three maximal isokinetic contractions with a 1 min rest between trials. Isokinetic strength assessments were performed at an angular velocity of 60°·sec^−1^ through a 70° ROM (20°–90°, where 0° is full knee extension). Peak isokinetic torque values were defined as the average of the highest attainable values for each of the three trials.

### Soreness

Soreness was also evaluated using a visual analog scale (VAS). A 100 mm line with 0 mm indicating “no pain” and 100 mm indicating “unbearable pain” was used. Subjects were first instructed to perform two hip/knee flexion and extensions (sitting and rising from a chair). The chair height was adjusted for each subject so that contact with the chair was made at a knee flexion angle of 90°. During the task subjects were asked to quantify the level of pain experienced in the knee extensor muscles and mark the VAS with a single vertical line accordingly. The distance from the left end of the scale to the mark was taken as the soreness level. All subjects provided a pre-exercise VAS evaluation.

### Immunohistochemistry

Eight-micrometer cross sections of muscle biopsy tissue samples were cut using a cryostat at −25°C. Samples were mounted to Superfrost slides and air-dried for 30 min. For slides that were stained for Pax7/type I myosin heavy chain (MyHC), Xin/Dystrophin, or tenascin C, sections were fixed in 2% paraformaldehyde (Sigma-Aldrich) for 4–8 min. Following fixation, sections were permeabilized in 0.2% Triton X-100 for 10 min and then blocked in a 2% bovine serum albumin (BSA), 5% fetal bovine serum (FBS) solution for 60 min at room temperature. Sections were incubated in the first primary antibody (Pax7, Xin, or tenascin C) in a humidified chamber overnight at 4°C. Following several washes, sections were then incubated in the appropriate secondary antibody for 30 min at 37°C. For double stained preparations, sections were then fixed and blocked again as described above and incubated with the second primary antibody (MyHC or dystrophin) for 1 h at RT. Following multiple washes, slides were incubated in DAPI, and the appropriate secondary antibodies for 30 min at 37°C. Stained slides were then dried and mounted using Fluoroshield histology mounting medium (Sigma–Aldrich). For MyoD staining, sections were fixed and permeabilized as described above (2% paraformaldehyde and 0.2% Triton X-100). The samples were then washed in PBST and incubated for 15 min in Avidin/Biotin blocking solution (Vector Laboratories, Burlingame, CA). A blocking solution consisting of 1% BSA, 2.5% Fetal Bovine Serum, 10% Normal Goat Serum, was then applied for 1 h at RT. The sample was then placed in a humidified chamber at 4°C and left to incubate overnight in MyoD1 antibody diluted 1:50 in 1% BSA. The following day the slides were washed in PBST and incubated in Anti-Mouse Biotin (Vector Laboratories, Burlingame, CA) at a ratio of 1:200 in 1% BSA for 1 h at RT. Following another wash cycle, sections were incubated in Rhodamine Red Streptavidin (1:1000; Jackson ImmunoResearch Laboratories) in 1% BSA and DAPI. Slides were covered for 30 min at RT. Stained slides were washed in PBST, dried, then mounted using Fluoroshield histology mounting medium (Sigma–Aldrich). Sections were imaged on an Olympus IX73 fluorescence capable inverted microscope. All antibody staining was verified using negative controls devoid of primary antibody. Human primary myoblasts cultured in a growth medium served as a positive control for the MyoD stain. The following primary antibodies were used: Pax7, IgG1 (cell supernatant 1:100; Developmental Studies Hybridoma Bank), myosin heavy chain I, IgG2b (1:100; Developmental Studies Hybridoma Bank; BA-D5), dystrophin, rabbit polyclonal (1:100; Abcam: ab15277), Xin, chicken polyclonal (1:500; a gift from Dr. Thomas Hawke, McMaster University), and MyoD, IgG1 (1:100; Dako; M3512), Tenascin C, rabbit polyclonal (1:100; Millipore: AB19013). Secondary antibodies used were: DyLight 488 IgG1 specific (1:100; Jackson ImmunoResearch Laboratories), Alexa Fluor 594 IgG2a specific (1:100; Jackson ImmunoResearch Laboratories), Alexa Fluor 594 IgG2b (1:100; Jackson ImmunoResearch Laboratories), Cy3 goat anti-rabbit (1:100 Jackson ImmunoResearch Laboratories), Alexa Fluor 488 goat anti-chicken (1:200 Abcam).

### Quantification of immunofluorescent images

Quantification of immunofluorescent images was carried out by an investigator that was blind to both condition and time point. For pax7/MyHC analyses, enumeration was made using 4 randomly acquired fields from all subjects at all time points. Images were taken using a 20× objective. An average of 425 ± 92 muscle fibers were analyzed per subject per time point. For MyoD analysis, enumeration of MyoD positive nuclei was made from 10 random fields acquired using a 40× objective from each sample at each time point. An average of 118 ± 24 muscle fibers were analyzed per subject per time point for the MyoD quantification. Quantification of tenascin C was carried out by calculating the total immunoreactive area of the entire section (~2–4 10× images that were taken at the same exposure time). For quantification, the total tenascin C immunoractive area was expressed relative to the total area of the imaged section. All analyses were done using Olympus cellSens software. An investigator that was blind to both time point and mode of contraction carried out imaging and quantification of all samples.

### Elisa-based MyoD activation assay

Due to limited protein from small biopsy samples from one individual in the CON group, we were only able to carry out ELISA-based MyoD analysis and cytokine profiling (see following section Cytokine Magnetic Bead Multiplex) in 6/7 subjects from this group. MyoD DNA binding activity was determined using nuclear extracts and an ELISA-based TransAM MyoD assay kit (Active Motif) according to the manufacturer's instructions. Nuclear protein was isolated using the NE-PER® Nuclear and Cytoplasmic Extraction kit (Thermo Scientific, Rockford, IL) according to the manufacturer's instructions. 5 μg of nuclear extract were added to wells coated with a consensus MyoD binding sequence and incubated for 1 h at room temperature. Wells were then washed and a primary antibody directed at MyoD was added and left to incubate for 1 h. This was followed by treatment of all wells with a secondary antibody conjugated to horseradish peroxidase (HRP). A subsequent colorimetric reaction was initiated with the addition of a developing solution for 5 min followed by the application of a stop solution. The absorbance of the plate was then read at 450 nm on a multi-well microplate reader (Victor 3; Perkin-Elmer, Waltham, MA). Wild type and mutated consensus oligonucleotides were used as competitors for MyoD binding to ensure specificity of the reaction as per the manufacturer's instructions. All samples were run in triplicate and the average value was used for data analysis.

### Cytokine magnetic bead multiplex

Cytokine Multianalyte profiling of cytoplasmic protein extracts from muscle biopsy samples were carried out on a Luminex Magpix multiplexing platform (Luminex Corporation, Austin, TX). Multiplexed cytokines were assessed using a human cytokine/chemokine 29-plex bead panel (Millipore Corporation, Billerica, MA). Cytokines that were analyzed included: VEGF, EGF, Eotaxin, G-CSF, GM-CSF, IFN-α2, IFN-γ, IL-1α, IL-1β, IL-1ra, IL-2, IL-3, IL-4, IL-5, IL-6, IL-7, IL-8, IL-10, IL-12 (p40), IL-12 (p70), IL-13, IL-15, IL-17, IP-10, MCP-1, MIP-1α, MIP-1β, TNF-α, TNF-β (see Supplemental Table 1). Mutiplexing analysis was performed using reagents supplied by Millipore according the manufacturers recommendations. Briefly, antibody-conjugated magnetic beads were incubated with 25 μg of tissue homogenate overnight at 4°C. Bead-complexes were then washed and incubated in biotinylated detection antibody for 30 min. on a plate shaker at RT. This was followed by incubation in streptavidin-phycoerythrin for 15 min. on a plate shaker at RT. Bead-complexes were then read on a Magpix multiplex platform (Luminex Corporation, Austin, TX). Median fluorescent values recorded from a minimum of 80 beads were used for data analysis. Sensitivity of standards ranged between 3.2 and 10,000 pg/ml, giving a broad range of sensitivity. Standard curves and data analysis was performed using Milliplex Analyst 5.1 software (Millipore Corporation, Billerica, MA).

### Statistical analysis

All measures except exercise performance comparisons were analyzed using a Two-Way repeated-measures ANOVA with factors for time (pre-exercise and post-exercise) and contraction mode (eccentric and concentric). Exercise performance data were analyzed using un-paired *t*-tests. All analyses were done using graphpad prism 6 software. Statistical significance was accepted at *p* < 0.05. Where significant main effects were found, Tukey's HSD *post hoc* tests were applied. Unless otherwise noted, data are presented as means ± *SD*.

## Results

### Subject characteristics and exercise performance

Subject characteristics and exercise performance data for the group that performed eccentric contractions (ECC) and the group that performed concentric contractions (CON) are presented in Table 1. There were no significant differences between ECC and CON for anthropometric measures. Likewise there was no difference in the amount of total work completed for ECC or CON. Because average peak torque was 44% greater for ECC compared to CON, individuals in CON performed a significantly higher number of maximal muscle contractions to maintain an equivalent workload. We noted significant variability that existed in CON due to disparities in the strength of subjects in that group. Over the course of the exercise protocol only CON experienced significant decreases in average peak torque achieved during each 2 kJ set, indicating greater fatigue in this group.

### Functional measures

**Figure 1:**
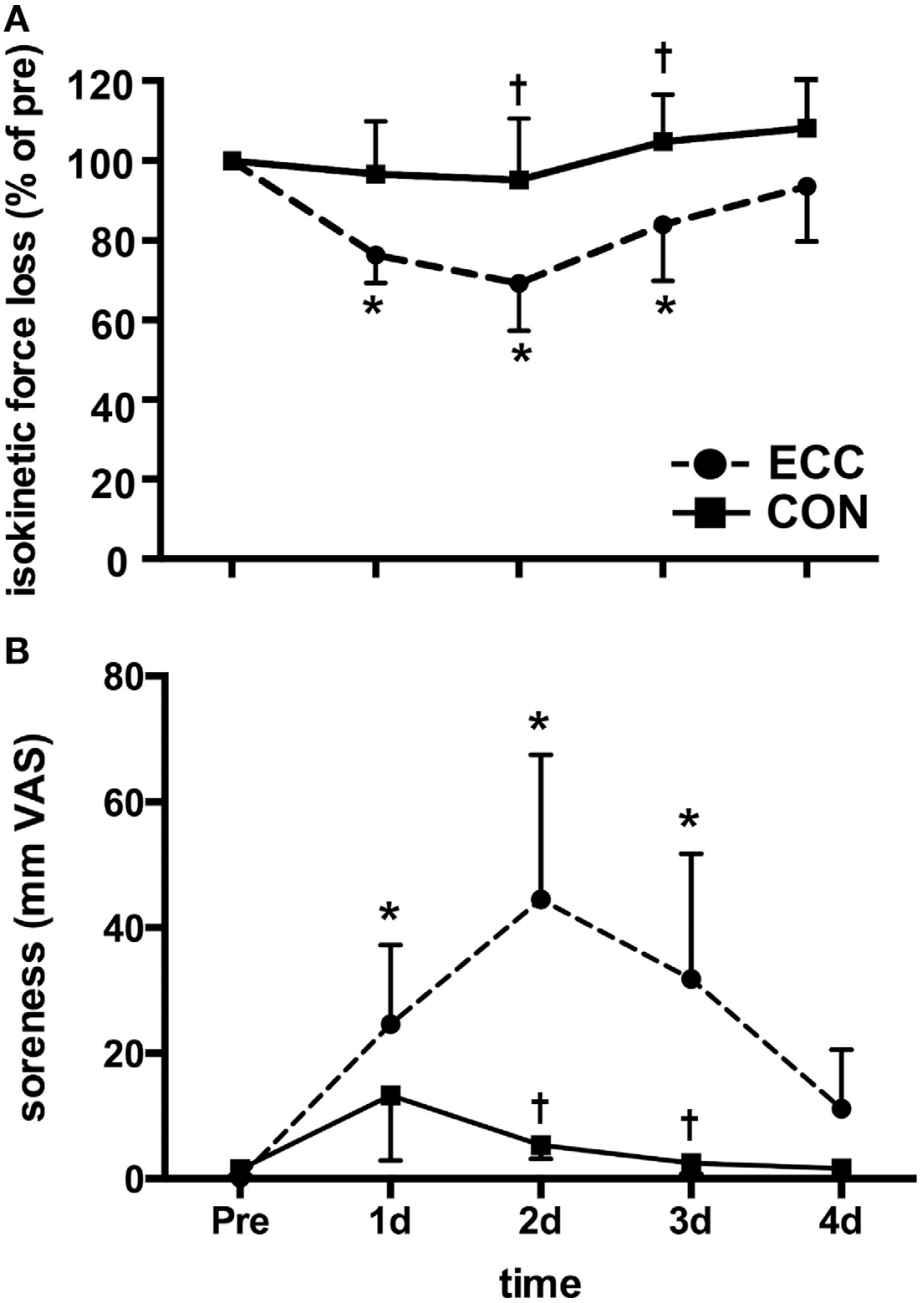
**Prolonged isokinetic strength loss and muscle soreness indirectly indicates mild muscle damage following eccentric (ECC) but not concentric contractions (CON)**. **(A)** Mean isokinetic strength of the knee extensors in both ECC and CON groups pre-exercise, and each day for 4 days following exercise. Data are presented as a percentage of pre-exercise force production. **(B)** Mean muscle soreness of the knee extensor muscles in both ECC and CON groups pre-exercise, and each day for 4 days following exercise. Data are presented as mm on a 100 mm visual analog scale (VAS). Data are means ± *SD*. ^*^Indicates significant difference (*p* < 0.05) from pre-exercise value. †Indicates significant difference between ECC and CON (*p* < 0.05).

Isokinetic peak torque values were analyzed and are presented in Figure 1 as percentages of the baseline values. Two-Way repeated measures ANOVA revealed significant (*p* < 0.01) effects of time, contraction mode, and the interaction for both isokinetic peak torque and muscle soreness measures (Figure 1). Isokinetic peak torque was significantly decreased relative to pre-exercise strength in ECC for 3 days post-exercise. Isokinetic strength loss peaked at 2 days post-ECC (33 ± 12%) (Figure 1A). There were no significant losses in force production at any time post-CON. ECC resulted in greater isokinetic force losses than CON at 2 and 3 days post-exercise. Muscle soreness increased significantly over time following ECC (*p* < 0.01), peaking at 2 days post-exercise (44.5 ± 9.4 mm VAS), and returning to baseline by day 4 (Figure 1B). There were no significant changes in muscle soreness following CON (Figure 1B). ECC resulted in significantly greater soreness compared to CON at 2 and 3 days post-exercise (*p* < 0.01).

### Myofiber damage and regeneration

**Figure 2:**
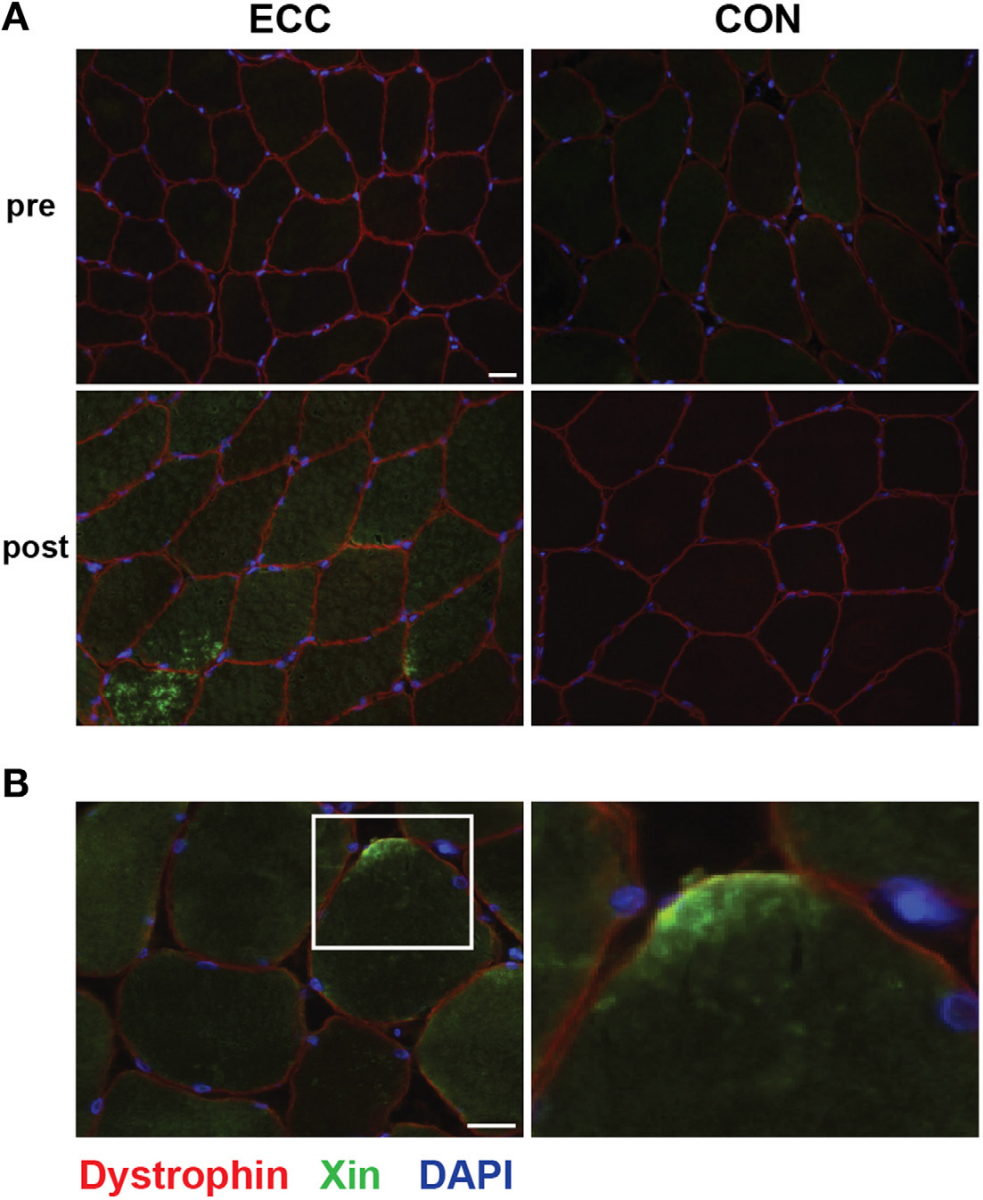
**Increased immunoreactivity and sarcolemmal localization of Xin suggests myofiber damage 24 h following eccentric (ECC) but not concentric contractions (CON)**. **(A)** Representative fluorescent images of a triple stained 8 μm section from pre- and 24 h post-exercise ECC and CON samples show increased immunoreactivity of Xin 24 h post-ECC. Scale bar = 20 μm. **(B)** Image of post-exercise ECC sample demonstrating the localization of Xin to the sarcolemma. White square in image denotes the boundaries of the enlarged image. Scale bar in merged image = 20 μm; scale bar in enlarged images = 10 μm.

To characterize myofiber damage, we immuno-stained muscle cross-sections for the recently characterized marker of muscle damage Xin (Figure 2). Consistent with the findings of Nilsson et al. (2013), Xin immunoreactivity was not detectable in any of the pre-exercise muscle cross-sections. In our qualitative analysis, we observed clearly increased Xin immunoreactivity post-exercise in all ECC biopsies, and 1/7 CON biopsies (Figure 2A). Evidence of Xin membrane localization was also present in the post-exercise ECC muscle (6/7 biopsies) more than CON muscle (2/7 biopsies; Figure 2B). As expected, due to the early time point post-exercise, we found no evidence of ongoing regeneration in either the ECC or CON post-exercise samples determined by the presence of central nuclei.

### Cytokine profile analysis

**Figure 3:**
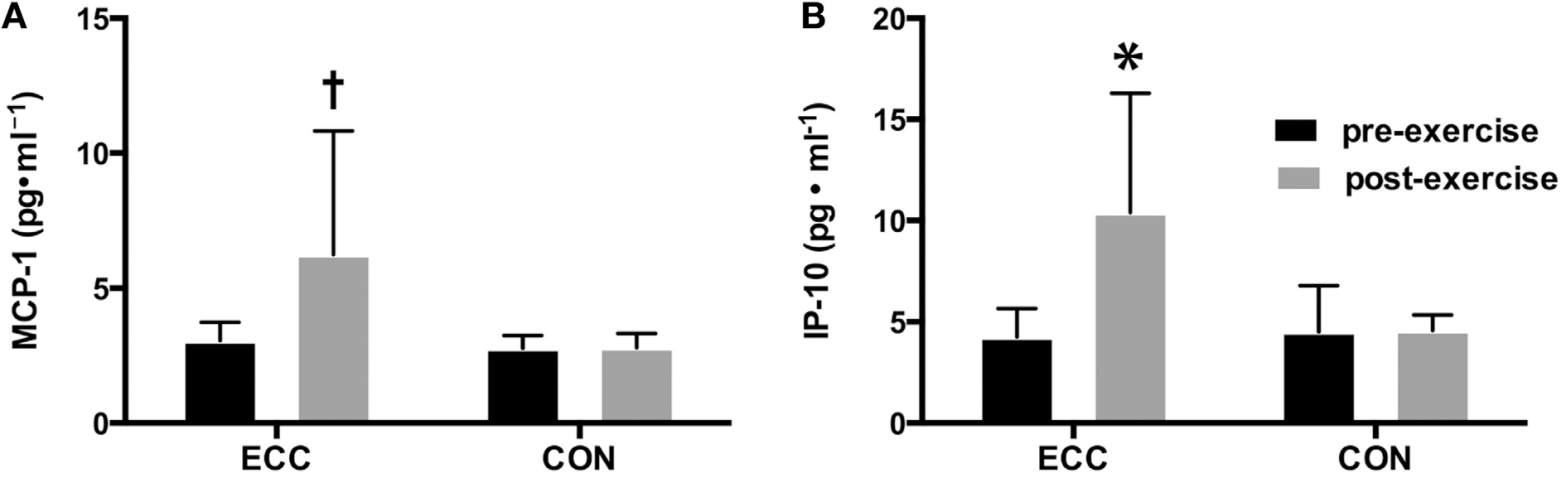
**Changes in pro-inflammatory cytokines following eccentric (ECC) but not concentric contractions (CON)**. Changes in MCP-1 **(A)** and IP-10 **(B)** concentrations pre- and 24 h post- exercise in muscle from subjects that performed a bout of ECC or CON. Data are means ± *SD*. ^*^Indicates significant difference (*p* < 0.05) from all other conditions. †Indicates significant difference (*p* < 0.05) between pre- and post-exercise measures.

As another measure of skeletal muscle damage, we assessed changes in inflammatory cytokine concentrations in biopsy homogenates using a cytokine multiplex assay. Overall, 17 of the 29 measured cytokines were found to be at concentrations at or below the lowest standard (3.2 pg/ml) in the majority of subjects. Those cytokines were therefore omitted from the analysis. Of the 12 cytokines that were analyzed, we found significant main effects for chemokine (C-C motif) ligand 2 (CCL2), also known as monocyte chemotactic protein-1 (MCP-1), and C-X-C motif chemokine 10 (CXCL10), also known as interferon gamma-induced protein 10 (IP-10). The concentration of IP-10 was increased following ECC, but not CON, showing significant main effects for time, contraction mode, and the interaction (*p* < 0.05) (Figure 3A). MCP-1 also increased following ECC, with a significant main effect of time (*p* < 0.05), and a statistical trend toward significance for contraction mode (*p* = 0.10) and the interaction (*p* = 0.079) (Figure 3B).

### Tenascin C

**Figure 4:**
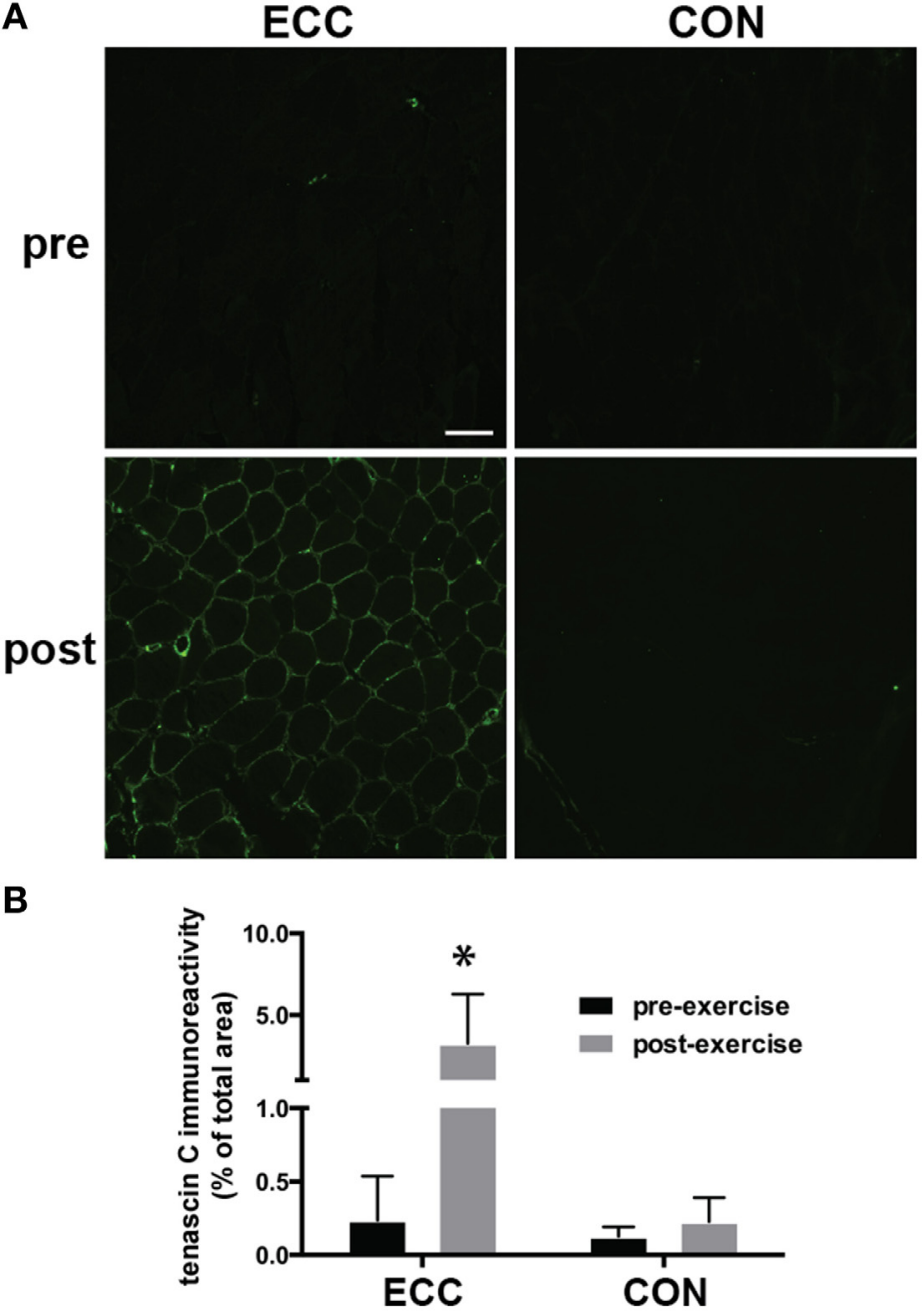
**Increased immunoreactivity of tenascin C suggests extracellular matrix (ECM) de-adhesion 24 h following eccentric (ECC) but not concentric contractions (CON)**. **(A)** Representative fluorescent images of pre- and 24 h post-exercise ECC and CON samples. Scale bar = 100 μm. **(B)** Quantification of tenascin C immunoreactivity pre- and 24 h post-exercise for ECC and CON. Data are means ± *SD*. ^*^Indicates significant difference (*p* < 0.01) from all other conditions.

To assess extracellular matrix (ECM) de-adhesion and remodeling, we measured immunoreactivity of the ECM de-adhesion protein tenascin C (Figure 4). The percentage of total muscle area that was immunoreactive for tenascin C increased from 0.2 ± 0.02% to 3.3 ± 3.0% pre- to 24 h post-ECC and was unchanged pre- (0.1 ± 0.06%) to 24 h post-CON (0.2 ± 0.1%) (Figures 4A,B). Two-Way repeated measures ANOVA showed significant effects of time (*p* < 0.01), mode (*p* < 0.01), and the time × mode interaction (*p* < 0.05).

### Total and fiber type specific satellite cell quantity

**Figure 5:**
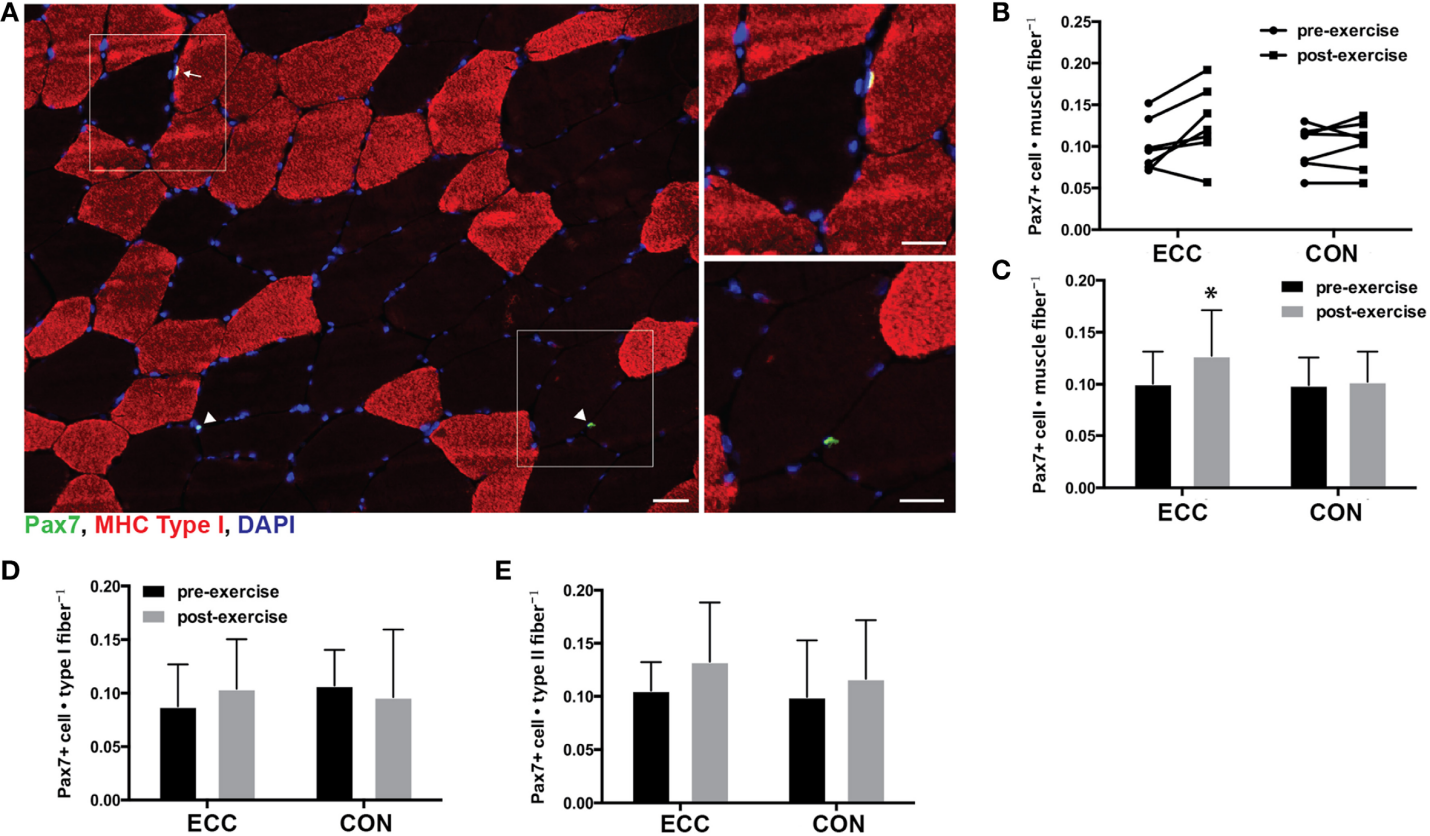
**Increased satellite cell content 24 h following eccentric (ECC) but not concentric contractions (CON). (A)** Representative fluorescent image of a triple stained 8 μm section from an ECC sample for type I myosin heavy chain (red), Pax7 (green), and nuclei (DAPI; blue). White squares in merged images denote the boundaries of the enlarged images. Arrow points to type I associated satellite cell and arrowhead points to type II associated satellite cells. Scale bar in merged image = 50 μm; scale bar in enlarged images = 20 μm. **(B)** Quantification of Pax7 positive nuclei for individual subjects pre- and post-exercise for both ECC and CON. **(C)** Mean satellite cell data presented as the total number of Pax7^+^ cells per muscle fiber. **(D)** Number of Pax7^+^ nuclei associated with Type I fibers pre- and post-exercise for ECC and CON. **(E)** Number of Pax7^+^ nuclei associated with Type II fibers pre- and post-exercise for ECC and CON. Data are means ± *SD*. ^*^Indicates significant difference (*p* < 0.05) between pre- and post-exercise measures.

For total mixed fiber satellite cell content, ANOVA revealed a significant effect of time (*p* = 0.028), but not mode or the time × mode interaction. Pre- to post-exercise, total satellite cell content per muscle fiber increased in ECC (0.101 ± 0.031 vs. 0.127 ± 0.041, respectively), but not CON (0.099 ± 0.027 vs. 0.102 ± 0.029, respectively) (Figures 5A–C). Also of note, total satellite cell number increased in all but 1 study participant in the ECC group (Figure 5B). As recent studies have shown that human skeletal muscle satellite cells respond to exercise in a fiber type specific manner 24 h following eccentric contractions (Cermak et al., 2013), we also determined how fiber type affected satellite cell proliferation in response to ECC and CON. We found no differences in the number of type I fiber-associated satellite cells pre- to post-exercise for ECC (0.08 ± 0.039 vs. 0.104 ± 0.046) or CON (0.107 ± 0.33 vs. 0.096 ± 0.063) (Figure 5D). Likewise we found no differences in the number of type II fiber-associated satellite cells pre- to post-exercise for ECC (0.106 ± 0.027 vs. 0.133 ± 0.056) or CON (0.10 ± 0.053 vs. 0.117 ± 0.055) (Figure 5E).

### MyoD activity

**Figure 6:**
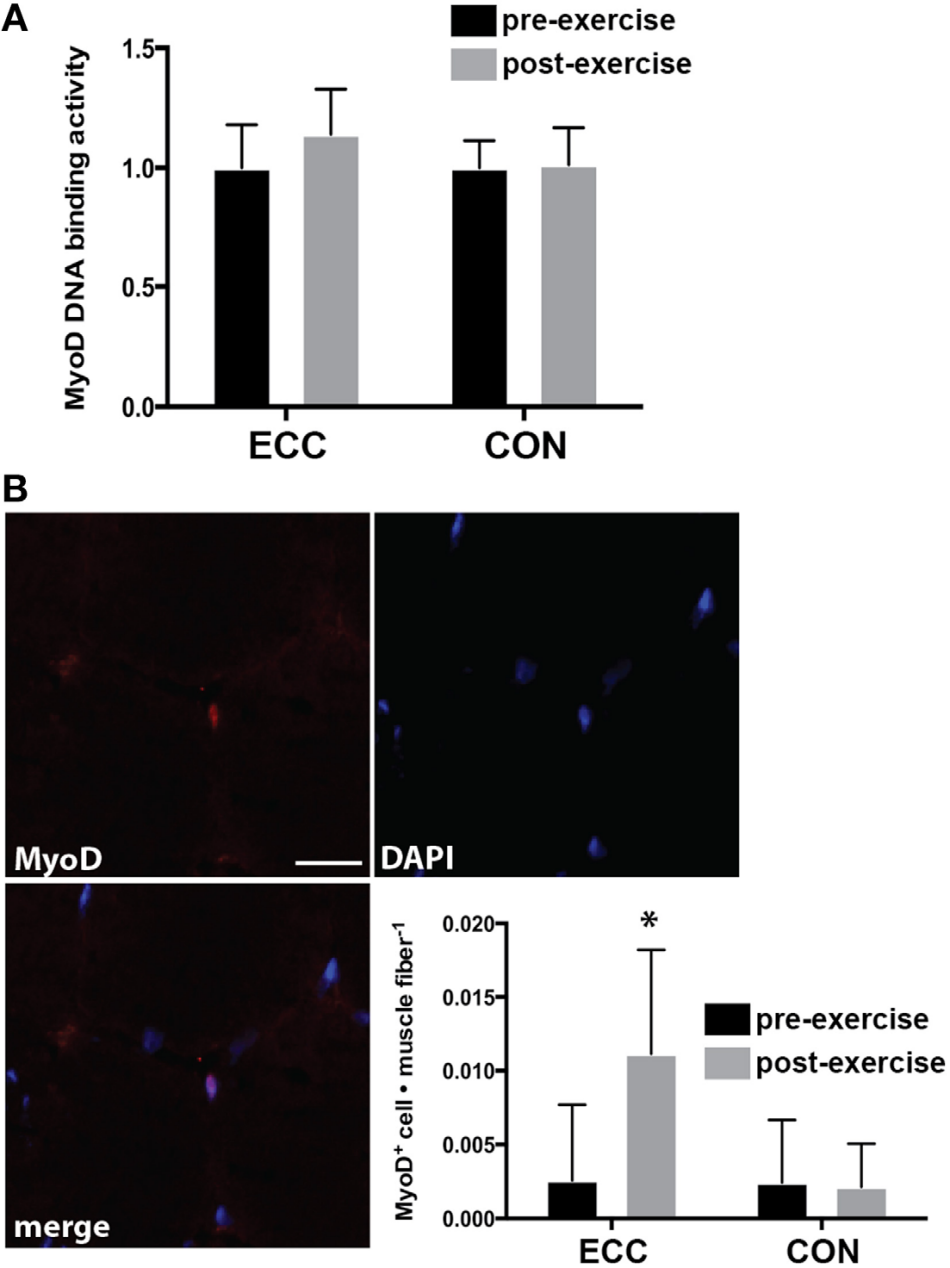
**DNA binding activity and nuclear accumulation of the myogenic regulatory factor MyoD 24 h following eccentric (ECC) or concentric contractions (CON)**. **(A)** DNA binding activity of MyoD from nuclear extract of muscle samples taken pre- and 24 h post-exercise for ECC and CON. **(B)** Image demonstrating a MyoD^+^ nucleus from a post-exercise ECC sample; and quantification of MyoD^+^ nuclei for subjects pre- and post-exercise for both ECC and CON. MyoD data are presented as the total number of MyoD^+^ cells per muscle fiber. Scale bar = 20 μm. Data are means ± *SD*. ^*^Indicates significant difference (*p* < 0.05) from all conditions.

The transcription factor MyoD is expressed in activated, but not quiescent satellite cells and represents one of the earliest markers of myogenic commitment. To assess the activity level of satellite cells following exercise, we quantified MyoD DNA binding activity and nuclear localization of MyoD in immunofluorescent sections. There were no changes in MyoD DNA binding activity pre- to post-exercise for ECC or CON as assessed by a transcription factor ELISA (Figure 6A). Nevertheless, a statistically relevant trend was noted for the main effect of time (*p* = 0.10). When we examined MyoD immunoreactivity in muscle cross sections, we found MyoD^+^ cells in only 5 of 14 pre-exercise biopsies (3 from ECC and 2 from CON), whereas we found MyoD^+^ cells in all biopsies post-ECC and in only 3 out of 7 biopsies post-CON (Figure 6B). MyoD^+^ cells per myofiber increased pre- (0.003 ± 0.005) to post-ECC (0.011 ± 0.007) and was unchanged pre- (0.002 ± 0.004) to post-CON (0.003 ± 0.003) (Figure 6B). Two-Way repeated measures ANOVA analysis of MyoD^+^ cells per myofiber revealed a significant effect of time (*p* < 0.05), mode (*p* < 0.05), and the interaction (*p* < 0.05).

## Discussion

The primary aim of the present study was to determine how contraction mode, when matched for total work, influenced indices of muscle damage and satellite cell activity in humans. Our primary findings were that a single bout of maximal eccentric (ECC), but not concentric (CON) muscle contractions induced: (1) muscle damage and evidence of ECM de-adhesion, and (2) an acute increase in the activation and quantity of mixed muscle fiber satellite cells 24 h post-exercise. This study confirms the longstanding notion that muscle damage primarily occurs due to ECC, but not CON (Friden et al., 1986; Faulkner et al., 1993), and is, to our knowledge, the first to show that exercise contraction mode influences satellite cell proliferation in human skeletal muscle following a single exercise bout.

Eccentric muscle contractions have been widely shown to be a strong stimulator of muscle satellite cell activity, which we define as the expression of MRFs (i.e., MyoD and myogenin) and re-entrance into the cell cycle (i.e., proliferation). Time course studies in human muscle show a steady expansion of the satellite cell pool from 24- to 72 h post-exercise that remains elevated above baseline for up to 8 days (Crameri et al., 2004a; O'Reilly et al., 2008; McKay et al., 2009, 2010; Cermak et al., 2013; Macaluso et al., 2013). In the present study we found an increased number of mixed muscle fiber satellite cells following eccentric exercise. This finding is in accordance with some (Dreyer et al., 2006; O'Reilly et al., 2008; McKay et al., 2009, 2010), but not all (Cermak et al., 2013) studies that have measured satellite cell quantity 24 h post exercise. Notably, the Cermak et al. study showed a significant increase in type II associated, but not total mixed fiber satellite cell quantity 24 h post-ECC. While numerous studies have detailed the expansion of the satellite cell pool post-eccentric contractions, the present study is the first to report a lack of satellite cell proliferation acutely (24 h) following a comparable bout of maximal concentric contractions, suggesting that changes in the satellite cell pool in the days following a typical bout of resistance exercise may be driven by the eccentric phase of those exercises. Interestingly, a recently published study reported that 12 weeks of concentric-only resistance training increased the satellite cell pool, whereas 12 weeks of eccentric-only training did not (Farup et al., 2014). Thus, it appears that CON is indeed capable of inducing satellite cell proliferation with chronic exposure and that the acute satellite cell response cannot predict the outcome of repeated loads.

In agreement with our satellite cell quantification, we found no change in DNA binding activity or nuclear accumulation of MyoD pre- to post-CON. In contrast, we found that ECC induced MyoD nuclear accumulation (assessed by immunofluorescence analysis). However, no change in MyoD DNA binding activity (measured by a DNA binding ELISA) was observed. Given that DNA binding activity was trending toward a difference pre- to post-exercise (*p* = 0.10), we suspect that we may have been underpowered to detect differences, as we originally powered the study based on the likelihood of finding significant increases in total satellite cell number, not MyoD DNA binding activity. Nevertheless, in accordance with our findings, those studies that have evaluated nuclear accumulation of MyoD in human muscle cross sections have found a statistically significant increased number of MyoD^+^ cells 24 h following exercise (McKay et al., 2012; Snijders et al., 2014). Notably, compared to the two cited studies, the total number of MyoD^+^ cells identified per muscle fiber in the current study was low. However, a direct comparison is difficult given that the two cited studies used a resistance training exercise that was different that the isolated eccentric exercise used in the current study. More studies are clearly needed to determine the extent of MyoD activity and its contribution to satellite cell proliferation following exercise in human muscle.

As previously established (Friden et al., 1986; Faulkner et al., 1993), our survey of whole muscle function and histological markers demonstrates the presence of damage following ECC, but not CON. However, unlike many previous studies, we carefully matched the total work of the exercise bout to account for the greater force producing capacity of ECC. In terms of loss in muscle force producing capacity relative to baseline, ECC, but not CON resulted in force losses post-exercise. Force losses in ECC were consistent with a moderate level of muscle damage when evaluated using criteria established by Paulsen et al. (2012). This was confirmed using immunohistological staining of Xin (Nilsson et al., 2013). It is worth noting that while we carefully matched the total work between the two modes of contraction, ECC exercised at longer muscle lengths than CON. The ROM difference during exercise may be of consequence because muscle damage is related to the amount of active muscle strain during exercise in animals (Lieber and Friden, 1993). In humans, markers of damage appear to be more pronounced at longer muscle lengths in the forearm flexors (Nosaka and Sakamoto, 2001). However, the relationship between muscle length and muscle damage in the knee extensors of humans is more uncertain, as no studies have directly addressed this question. While we are unaware of any studies that have directly examined how muscle length affects damage induced by CON, we cannot rule out the possibility that the exercise at shorter muscle lengths in CON may have affected the results.

In this study we verified the presence of muscle damage directly via the immunoreactivity of the striated muscle-specific protein Xin and the presence of inflammatory cytokines. Nilsson et al. (2013) recently demonstrated that Xin immunoreactivity was positively correlated to the degree of muscle damage in an array of myopathies. Furthermore, Xin immunoreactivity was shown to increase 24 h following a damaging eccentric exercise, which made it an ideal marker for our single 24 h sampling time point (Nilsson et al., 2013). Consistent with that report, our qualitative analysis of Xin stained cross-sections revealed a diffuse increase in Xin immunoreactivity in the eccentrically exercised myofibers. Furthermore, we observed high levels of Xin localization at the sarcolemma in the majority of the eccentrically exercise samples. Conversely, Xin was virtually undetectable in all the pre-exercise samples and the post-exercise samples of concentrically exercised muscle, suggesting a lack of myofiber damage in these conditions. As another direct indicator of muscle damage, we probed for changes in inflammatory cytokines using a panel of 29 cytokines/chemokines in a multiplexed magnetic bead assay. Surprisingly, we found statistically significant changes in only 2 of the cytokines/chemokines (MCP-1 and IP10) following ECC and no changes in any of the measured cytokines/chemokines following CON. Several previous studies have identified increases in MCP-1 in muscle (Hubal et al., 2008; Catoire et al., 2014) and in the circulation (Crystal et al., 2013; Catoire et al., 2014) following various forms of exercise and have suggested a role for MCP-1 in recruiting inflammatory cells and as a autocrine/paracrine factor involved in cross-talk between muscle and other organs. Our observation that IP-10 increases following ECC is novel. There are a limited number of studies on the potential functions of IP-10 in skeletal muscle, but most available literature suggest its involvement in the pathogenesis of inflammatory myopathies and the recruitment of T lymphocytes (Dufour et al., 2002; Feferman et al., 2005; Crescioli et al., 2012). Interestingly, a recent study showed that skeletal muscle T-cell infiltration enhances satellite cell activity and improves muscle regeneration in mice (Burzyn et al., 2013), which, taken together with our data, suggests a potential role for IP-10 as a mediator of satellite cell activity and muscle regeneration. Collectively, the cytokine data point to a mild pro-inflammatory response suggestive of muscle damage following ECC but not CON.

We also extended our analysis of damage beyond the myofiber to changes occurring in the extracellular matrix (ECM). Certainly one of our most robust findings was the contraction mode dependency of tenascin C expression. While tenascin C immunoreactivity was clearly increased in the ECM post-eccentric exercise, its absence was equally apparent post-concentric exercise. Tenascin C expression has been shown to increase following both eccentric contractions and damage induced by electrical stimulation in human muscle (Crameri et al., 2004b; Raastad et al., 2010; Mackey et al., 2011). However, its response to mechanical load under lower strain conditions (isolated concentric exercise) had not previously been evaluated. The role of tenascin C in skeletal muscle is believed to be the disassembly of focal adhesion complexes and general de-adhesion of the ECM (Murphy-Ullrich, 2001). The coordinated de-adhesion of the ECM structure is thought to provide relief from strain and promote regeneration by facilitating the expansion and motility of ECM-resident cells following mechanically-induced damage (Fluck et al., 2008; Murphy-Ullrich and Sage, 2014). Thus, the absence of changes in tenascin C immunoreactivity following concentric exercise suggests that this contraction mode, when performed in isolation, does not induce extensive ECM de-adhesion and subsequent remodeling. This assertion is supported by a previous study that demonstrated greater changes in ECM-related gene expression (TGF-β signaling and collagens) in response to eccentric relative to concentric contractions in rat muscle (Heinemeier et al., 2007). The differential effect of contraction mode on both satellite cell activity and tenascin C expression suggests that mechanically-induced ECM de-adhesion may influence the activity of ECM dwelling satellite cells. Indeed, a tenascin enriched matrix favors muscle regeneration *in vivo* and migration of primary myoblasts *in vitro* (Fluck et al., 2008; Calve et al., 2010; Mercer et al., 2013). Although more investigation is required, in light of our data, it is compelling to consider a cross-talk relationship between satellite cells and the muscle ECM.

A limitation to the present study is the single sampling point of 24 h post-exercise, inasmuch as robust changes in muscle satellite cell content occur at later time points (48–72 h post-exercise) (Crameri et al., 2004a; O'Reilly et al., 2008; McKay et al., 2009). It is possible that, in our subjects, significant satellite cell changes continued to occur in the days following our sampling time point in response to both contraction conditions. Likewise, it is possible that histological evidence of myofiber or ECM damage may have manifested additional information at later time points, providing evidence that would influence our current interpretation. The rationale for the single 24 h post-exercise sampling time point was to maximize our ability to detect early changes (markers of satellite cell activation), and the resultant effects (satellite cell proliferation) of those changes, while minimizing the invasiveness and potential confounding interactions of multiple muscle biopsies. Several prior studies have identified the 24 h post-exercise time point as the earliest time point to reliably detect changes in satellite cell number. Another limitation is the small sample size of 7 subjects per group. Our sample size likely contributed to the discrepant results between MyoD DNA binding activity (ELISA) and MyoD nuclear accumulation (IHC) as discussed above. Nevertheless, an *a priori* power analysis, using previously published data, predicted that 7 subjects per group would be sufficient to detect differences in satellite cell quantity 24 h post-exercise. Our data support that prediction. Nevertheless, future studies with greater statistical power and multiple sampling time points will be valuable in confirming and extending the findings of the present study.

In conclusion, we have shown that a bout of eccentric but not concentric contractions induces (1) evidence of both functional and histological muscle damage, and (2) activation and proliferation of satellite cells at an early time point (24 h) post-exercise. Furthermore, we show that contraction mode influences ECM de-adhesion following exercise. These data lead us to speculate that ECM de-adhesion may be an important event that regulates the activation and proliferation of muscle satellite cells. As recent studies have highlighted the importance of satellite cell activity for the growth and repair of skeletal muscle, the determination of exercise parameters, such as contraction mode, to optimize satellite cell activity may have significant clinical relevance.

## Author contributions

Robert D. Hyldahl and Allen C. Parcell contributed to the conception and design of the work, the acquisition, analysis and interpretation of the data, drafting of the manuscript, and final approval of the manuscript. Ty Olson, Tyson Welling, and Logan Groscost contributed to the acquisition and analysis of the data, and the drafting and final approval of the manuscript. All authors have agreed to be accountable for all aspects of the work related to its accuracy and integrity.

### Conflict of interest statement

The authors declare that the research was conducted in the absence of any commercial or financial relationships that could be construed as a potential conflict of interest.
