# Supplementary material for: Satellite cell activity is differentially affected by contraction mode in human muscle following a work-matched bout of exercise
Source: Front Physiol. 2014 Dec 11;5:485. doi: 10.3389/fphys.2014.00485 (PMC4263080; doi:10.3389/fphys.2014.00485)
Supplement: Supplementary file 1 [file Table1.docx]

***Supplementary Material***

**Satellite cell activity is differentially affected by contraction mode in human muscle following a work-matched bout of exercise**

**^1^Robert D. Hyldahl, ^1^Ty Olson, ^1^Tyson Welling, ^1^Logan Groscost, ^1^Allen C. Parcell**

1. Brigham Young University, Department of Exercise Sciences, Provo, UT, USA

*** Correspondence:** Robert D. Hyldahl, Brigham Young University, Department of Exercise Sciences, 106 Smith Fieldhouse, Provo, UT, 84604, USA.

[robhyldahl@byu.edu](mailto:robhyldahl@byu.edu)

1. **Supplementary Figures and Tables**

## Supplementary Tables

**Supplementary Table 1.** Cytokine concentrations in skeletal muscle biopsy samples pre- and 24h post- a single bout of maximal eccentric and concentric contractions. Table shows data for 12 out of 29 cytokines that were measured using a multiplexed magnetic bead assay. The concentration of the 17 remaining cytokines was below the detectable limit. They included: Eotaxin, Granulocyte-macrophage colony-stimulating factor (GMCSF), Interferon gamma (IFNγ), Interleukin 19 (IL10), Interleukin 12 p40 (IL12P40), Interleukin 12 p70 (IL12P70), Interleukin 17A (IL17A), Interleukin 1 receptor antagonist (IL1RA), Interleukin 1 alpha (IL1α), Interleukin 1 beta (IL1β), Interleukin 2 (IL2), Interleukin 3 (IL3), Interleukin 5 (IL5), Interleukin 8 (IL8), Macrophage inflammatory protein 1 beta (MIP1β), Tumor necrosis factor alpha (TNFα), Tumor necrosis factor beta (TNFβ). * indicates significant difference (p<0.05) from all other conditions. ^∞^ indicates significant difference (p<0.05) between pre- and post-exercise measures.

| **Cytokine** | **Abbreviation** | **Eccentric (ECC)** | | **Concentric (CON** | |
| --- | --- | --- | --- | --- | --- |
|  |  | **Pre (pg•ml^-1^)** | **24h post (pg•ml^-1^)** | **Pre (pg•ml^-1^)** | **24h post (pg•ml^-1^)** |
| Epidermal growth factor | EGF | 6.6 ± 0.9 | 6.2 ± 1.0 | 6.8 ± 1.3 | 5.8 ± 1.4 |
| Granulocyte colony-stimulating factor | GCSF | 5.2 ± 1.4 | 5.9 ± 1.3 | 2.8 ± 0.5 | 2.4 ± 0.4 |
| Interferon alpha 2 | IFNa2 | 2.5 ± 0.5 | 2.4 ± 0.8 | 2.8 ± 0.5 | 2.4 ± 0.4 |
| Interleukin 13 | IL13 | 9.1 ± 4.7 | 7.2 ± 2.9 | 8.6 ± 3.0 | 7.7 ± 2.4 |
| Interleukin 15 | IL15 | 5.0 ± 2.5 | 6.12 ± 5.5 | 7.2 ± 5.6 | 5.6 ± 3.3 |
| Interleukin 4 | IL4 | 7.5 ± 6.4 | 8.7 ± 7.4 | 18.5 ± 15.0 | 21.8 ± 13.4 |
| Interleukin 6 | IL6 | 3.1 ± 1.8 | 3.9 ± 1.6 | 5.2 ± 2.8 | 4.7 ± 2.2 |
| Interleukin 7 | IL7 | 2.5 ± 1.4 | 2.6 ± 1.5 | 3.1 ± 1.5 | 2.6 ± 1.2 |
| Interferon gamma-induced protein 10 | IP10 | 4.3 ± 1.4 | 10.4 ± 5.9* | 4.5 ± 2.3 | 4.5 ± 0.8 |
| Monocyte chemotactic protein 1 | MCP1 | 2.8 ± 0.5 | 6.2 ± 4.6^∞^ | 2.8 ± 0.5 | 3.1 ± 0.7 |
| Macrophage inflammatory protein 1 alpha | MIP1a | 4.7 ± 3.0 | 4.5 ± 2.8 | 8.0 ± 4.9 | 7.4 ± 5.6 |
| Vascular endothelial growth factor | VEGF | 6.9 ± 4.0 | 7.5 ± 3.6 | 15.1 ± 10.5 | 11.1 ± 3.8 |
